# Awake prone positioning effectiveness in moderate to severe COVID-19 a randomized controlled trial.

**DOI:** 10.12688/wellcomeopenres.22792.1

**Published:** 2024-09-20

**Authors:** Nguyen Thanh Phong, Du Hong Duc, Ho Bich Hai, Nguyen Thanh Nguyen, Le Dinh Van Khoa, Le Thuy Thuy Khanh, Luu Hoai Bao Tran, Nguyen Thi My Linh, Cao Thi Cam Van, Dang Phuong Thao, Nguyen Thi Diem Trinh, Pham Tieu Kieu, Nguyen Thanh Truong, Vo Tan Hoang, Nguyen Thanh Ngoc, Tran Thi Dong Vien, Vo Trieu Ly, Tran Dang Khoa, Abigail Beane, James Anibal, OUCRU COVID RESEACH GROUP, Guy E Thwaites, Ronald Geskus, David Clifton, Nguyen Thi Phuong Dung, Evelyne Kestelyn, Guy Glover, Le Van Tan, Lam Minh Yen, Nguyen Le Nhu Tung, Nguyen Thanh Dung, C Louise Thwaites

**Affiliations:** 1 Hospital for Tropical Diseases, Ho Chi Minh City, Vietnam; 2 Oxford University Clinical Research Unit, Ho Chi Minh City, Ho Chi Minh, Vietnam; 3 University of Medicine and Pharmacy Ho Chi Minh City, Ho Chi Minh City, Ho Chi Minh, Vietnam; 4 Mahidol Oxford Tropical Medicine Research Unit, Bangkok, Bangkok, Thailand; 5 Institute for Biomedical Engineering, Oxford, UK; 6 University of Oxford Centre for Tropical Medicine and Global Health, Oxford, England, UK; 7 Guys and St Thomas's Hospitals, London, UK

**Keywords:** awake prone position; low- middle- income country, randomised controlled trial, COVID-19

## Abstract

**Background:**

Awake prone positioning (APP) may be beneficial in patients with respiratory failure who are not receiving mechanical ventilation. Randomized controlled trials of APP have been performed during peak COVID-19 periods in unvaccinated populations, with limited data on compliance or patient acceptability. We aimed to evaluate the efficacy and acceptability of APP in a lower-middle income country in an open-label randomized controlled trial using a dedicated APP implementation team and wearable continuous-monitoring devices.

**Methods:**

The trial was performed at a tertiary level hospital in Ho Chi Minh City, Vietnam, recruiting adults (≥18 years) hospitalized with moderate or severe COVID-19 and receiving supplemental oxygen therapy via nasal/facemask systems or high-flow nasal cannula (HFNC). Patients were allocated by a computer-generated random number sequence in a 1:1 ratio to standard care or APP, where a dedicated team provided bedside support. Wearable devices continuously recorded pulse oximetry and body position continuously. Our primary outcome was escalation of respiratory support within 28 days of randomization.

**Results:**

Ninety-three patients were enrolled in this study between March 2022 and March 2023. Eighty (86%) patients had received ≥2 doses of SARS-CoV2 vaccine. The study was terminated early because of a reduction in the number of eligible patients. Data from 46 patients allocated to APP and 47 to standard care were available for analysis. At baseline, 19/47 (40%) patients allocated to the standard care group and 14/46 (30%) patients allocated to the APP group received HFNC. Continuous monitoring data were available for all patients monitored with wearable devices. Significantly greater mean daily APP times were achieved in those allocated to APP, however, most achieved less than the target 8 h/day. We did not detect clear differences in the primary outcome (relative risk,RR, 0.85, 95% CI 0.40-1.78, p=0.67) or secondary outcomes, including intubation rate and 28-day mortality. Patients reported prone positioning was comfortable, although almost all patients preferred supine positioning. No adverse events associated with the intervention were observed.

**Conclusions:**

APP was not associated with benefit, but there was no sign of harm. Continuous monitoring with wearable devices is both feasible and acceptable for patients. In our population, achieving prolonged APP time was challenging despite a dedicated support team, and patients preferred supine positioning.

**Clinical Trials Registration:**

NCT05083130.

## Plain language summary

Lying on ones front (prone) can have beneficial effects for the lungs in very sick patients on ventilators. In people not on ventilators, but nevertheless requiring breathing assistance through oxygen devices, it is less clear whether this position is still beneficial. There have been several studies in patients with COVID-19 examining this, but results differ between studies. Very few studies have been carried out in patients who have been vaccinated against COVID-19, or in low and middle income countries.

We carried out a study in Vietnam, a lower-middle income country to examine this. In total 93 patients participated in the study and were randomly treated following standard care (verbal advice to change positions) or were provided with a special team present in the ward to help them and encourage them to lie in a prone position for as long as possible every day. The rate of patients requiring next level of support for breathing was compared between groups.

Our results showed that there was no difference between treatment groups in the rate of patients requiring extra breathing support, or in mortality rates, although some indication of shorter hospitalization times in those lying prone. Participants reported that they found lying prone comfortable, however most still preferred to lie on their back.

## Introduction

In mechanically ventilated patients with acute respiratory distress syndrome (ARDS), prone positioning is associated with improved survival ^1^. The benefit of prone positioning in patients not receiving invasive mechanical ventilation is less clear, but the COVID-19 pandemic stimulated multiple randomized controlled trials of prone positioning in non-mechanically ventilated patients, termed awake prone positioning (APP). Comparison of these studies is enabled by the consistent use of similar endpoints and several studies using a harmonized protocol ^2^. However, conflicting results have been reported. While a meta-analysis showed the overall benefit of APP in patients with COVID-19, a subgroup analysis showed no benefit in those treated outside the ICU or where patients received lower levels of baseline respiratory support ^3^. A more recent non-randomized trial of 501 patients in the USA receiving supplemental oxygen for COVID-19 pneumonia, including non-ICU settings, reported worse outcomes in those allocated to APP, with patients requiring a higher level of oxygen support on day 5 ^4^. Differences in the reporting of the APP intervention itself, patient compliance with the intervention, and differences in comparator groups remain significant impediments to understanding these conflicting results. Nevertheless, a better understanding is vital to inform decision-making, resource allocation, and policy around APP, particularly in low-resource settings where limited staff already make implementing APP challenging ^5, 6^.

In ventilated patients, the duration of prone position is a key factor in determining its efficacy, and data from APP studies also indicate that this is important ^7^. Accurately quantifying the duration of an APP is difficult, particularly under pandemic pressure. While several studies have not reported APP duration, others have relied on nursing reports, electronic health records, or reports from patients themselves ^5, 8– 14^. In low-resource settings, these methods are usually unfeasible or unavailable outside ICUs.

Duration may also be confounded by disease severity and implementation methods. More severely ill patients are more likely to be cared for in settings with greater access to staff for support in turning and maintaining APP, whereas patients with lower oxygen requirements, cared for in less intensive environments, are more able to move themselves to a position of their choice. Studies in the UK, Pakistan, and China that have examined acceptability from patient perspectives suggest overall negative attitudes to APP related to discomfort, physical consequences, and social factors that may influence patients’ willingness to self-prone ^15– 17^. Concurrent patient perspectives from randomised controlled trials are lacking.

The aim of this study was to evaluate APP in an LMIC setting using a dedicated team to evaluate prone duration and assist patients with the allocated study intervention. While conceived at the height of the pandemic in Vietnam, our study was implemented after widescale population-level vaccination coverage in Vietnam and is, to our knowledge, the only randomized trial in a largely vaccinated population. To reduce the burden on staff, we introduced wearable monitors to facilitate remote patient monitoring. Our team has already developed monitoring with low-cost pulse oximeters, in which wearables are used for both vital sign monitoring and quantification of prone position duration by incorporating a low-cost accelerometer ^18^.

## Methods

### Trial design

This was an open-label, randomized controlled trial. A Trial Steering Committee oversaw the trial, and an independent Data and Safety Monitoring Review Board (DSMB) reviewed all severe adverse events and data for safety endpoints at pre-specified time points. The full protocol was published separately ^19^. The study was stopped before reaching the predetermined sample size because of the low number of cases and the infeasibility of reaching the proposed sample size.

### Ethics

The study protocol was approved by the Ethical Committee of the Hospital for Tropical Diseases and the Oxford Tropical Research Ethics Committee. All participants or their representatives provided written informed consent before enrolment in the study.

### Setting

This study was conducted at the Hospital for Tropical Diseases, Ho Chi Minh City. The hospital is a tertiary referral center for infectious diseases in southern Vietnam and a designated special treatment center for COVID-19 throughout the pandemic.

### Participants

Adult patients aged ≥18 years with a diagnosis of probable or confirmed COVID-19 were eligible for inclusion in the study if they had moderate or severe COVID-19 (Vietnam Ministry of Health criteria) and required supplemental oxygen ^19^. Patients who had already received noninvasive ventilation (continuous or bilevel positive airway pressure), mechanical ventilation, or with contraindication to prone positioning, body mass index > 35, pregnancy, Glasgow coma scale <13, or a decision not to escalate care were excluded from the study. All patients or their representatives provided written informed consent prior to enrollment.

### Intervention

Those in the standard care group received verbal and written instructions conforming to the Vietnamese Ministry of Health Guidelines, as well as visits by the study team at the beginning and end of possible proning times. These instructions include changing position between prone, supine, lateral, and semi-recumbent every two hours (See Supplementary materials). Patients in the APP group were visited by the study team and provided written and verbal advice about lying in the prone position, as well as assistance in achieving and maintaining a fully prone position for as long as possible. All study procedures were carried out by a specific study team who were present in the ward at 8am-5pm daily and dedicated ward nurses who supervised the evening APP session (6–8pm). Patients were followed up daily for study outcomes until hospital discharge or transfer. Day 28 outcomes were collected by telephone follow-up for patients who were already discharged from the hospital at this time.

### Randomisation

Patients were enrolled by the study staff prior to randomization. Enrolled patients were randomized in a 1:1 ratio to the two allocations according to a computer-generated random list using block randomization with variable block length to the standard care or APP groups. An independent statistician generated this list.

### Procedures

APP was initiated as soon as possible after randomization and continued until either escalation or cessation of oxygen therapy. Patients in the APP group were supported to be in the prone position for as long as possible while the study staff were attending the ward, except for mealtimes or other nursing procedures. Support included both physical assistance and physical aid such as pillows. No support was available to assist patients in turning prone at night time after 8pm. Routine management was provided by ward staff and followed the Vietnam Ministry of Health Guidelines. In addition, all patients underwent continuous SpO _2_ (SmartCare Analytics, UK) and accelerometry (Axivity AX3, Newcastle upon Tyne, UK) monitoring with wearable devices during the intervention period. The pulse oximeters were connected via Bluetooth to a bedside tablet where data could be visualized and transmitted to the cloud server for remote visualization and downloading of data ^20^. Accelerometers were attached to the patient’s infraclavicular fossa to infer prone vs. non-prone positions. The duration of prone position was also recorded manually by the study staff in the ward. To determine the acceptability of APP and wearable device monitoring, prior to hospital discharge, a questionnaire was administered to patients by trained study staff using a 10-point Likert scale to evaluate patient experiences (Protocol ^19^).

### Outcomes

The primary outcome was escalation of respiratory therapy within 28 days of randomization, defined as intubation or escalation to the next level of respiratory support (with lowest level nasal canulae or face mask, escalating from HFNC to NIV or mechanical ventilation).

Secondary outcome measures included the requirement for intubation and mechanical ventilation within 28 days of randomization, 28-day all-cause mortality, in-hospital mortality, duration of hospital stay, SpO _2_ /respiratory rate/ heart rate/ FiO2, and ROX index ([SpO _2_/FiO _2_]/respiratory rate) before and at the end of the period of prone positioning every day, duration of oxygen, and estimated oxygen consumption. Adverse events were monitored daily by the study team.

The sample size was calculated based on a treatment failure rate of 52%, and a relative risk of treatment failure of 0.8 for the intervention was calculated at a two-sided 5% significance level, leading to 300 patients in each arm.

### Statistical methods

Data analysis followed a *priori* defined statistical analysis plan completed before database locking ( https://doi.org/10.1101/2024.06.30.24309722).

The primary population for all analyses was the full population of all randomized patients. The patients were analyzed according to their randomized arms (intention-to-treat). Analyses for the primary endpoint were repeated on the per-protocol population, which excluded the following patients: patients not receiving the randomized intervention and other major violations of inclusion/exclusion criteria or study procedures.

The primary outcome measure was compared between the groups based on a logistic regression model, with the intervention as the only covariate. As odds ratios from logistic regression are somewhat difficult to interpret, we additionally estimated the RR between the groups based on a binary regression model with a log-link rather than the logit link function used in logistic regression. The heterogeneity of the treatment effect was not assessed because of the small sample size. For secondary dichotomous outcomes, such as requirement for intubation and mechanical ventilation within 28 days of randomization, we computed the number of patients who developed or did not develop the outcomes of interest and fitted a logistic regression.

For duration of hospital stay, ventilator-free days, time from enrolment to first escalation of respiratory therapy, time from enrolment to first intubation, hospital mortality was treated as a competing event. Time-to-event analysis was performed using a subdistribution hazards model. Cause-specific cumulative incidence was estimated and plotted. Differences between intervention groups was tested using Gray’s log-rank test. In-hospital mortality was assessed with both a logistic regression model and estimated via Kaplan-Meier curves and compared using the log-rank test. We assumed that individuals that did not die remained alive until day 90. Patients discharged for palliative care were considered in-hospital deaths.

For continuous outcomes, such as vital signs (SpO _2_ /respiratory rate/ heart rate/ FiO _2_/ROX index) (repeated measurements) before and at the end of prone sessions (morning and afternoon, excluding evening), interventions were compared using a linear mixed-effects model, with or without a quadratic term for both fixed and random effects if it gave the model a better fit (p<0.05).

### Wearable data preprocessing

Accelerometers were worn continuously and oximeters were worn during observation periods between 9am and 8am the following day. For accelerometry, 3 axis measures at a frequency of 100 Hz from the accelerometers were used to calculate the angular positions, defined by degrees in the x-plane (left-right from -180 to 180), y-plane (prone-supine -180 to 180 degrees), and z-plane (up-down from -180 to 180). To determine the’ prone' label for 30 s segments, we first smoothed the measures using the median for 30 s consecutive moving window; then, we applied a threshold of (|x| < 60, |y| < 40, |z| < 60) degrees. This threshold was obtained by a grid search for |x|, |y|, and |z| between 0 and 90, step 5 degrees, and optimized for the prone morning and afternoon sessions observed by the study staff.

### Adverse events

All adverse events, defined according to the Common Terminology Criteria for Adverse Events as “any untoward medical event that occurs to a study participant during the course of the study” and followed their grading (grade 1: mild to grade 4: severe) were recorded ^19, 21^. Serious adverse events were defined as those that were life-threatening or resulted in death, new inpatient hospitalization or prolongation of existing hospitalization, persistent or significant disability, or congenital anomalies. All serious and additional specified adverse events were reported to the study data monitoring and safety board and relevant ethical committees.

### Role of the funding source

The study funder had no role in the study design, data collection, data analysis, data interpretation, or writing of the manuscript.

## Results

Ninety-three patients were enrolled between 8 ^th^ March 2022 and 23 ^rd^ March 2023 and were followed up until 1 ^st^ May 2023. Despite the Hospital for Tropical Diseases remaining the dedicated COVID-19 treatment center for Ho Chi Minh City (population 10 million), a reduction in admissions with COVID-19 and eligible study patients meant that recruitment was significantly impacted. The Trial Steering Committee and Data Monitoring and Safety board approved the early cessation of the study owing to the unfeasibility of reaching the planned sample size, balanced with duty for timely reporting and sharing of already acquired data. Consequently, 46 and 47 patients were enrolled in the APP and standard care groups, respectively. Three patients were transferred to other wards before cessation of oxygen therapy and excluded from the per-protocol analysis.

The baseline patient characteristics are presented in Table 1. Eighty-five out of 93 (92%) patients had received at least one dose of COVID-19 vaccine, with 80/93 (86%) receiving two or more doses and 42/93 (45%) 3 or more doses. Patients received the allocated intervention for a median of 4.95 days (interquartile range (IQR) 3.0-7.8) in the standard care group and 3.94 days (IQR 2.9-7.2) in the APP group. Mean of daily APP duration per individual observed by study staff was a median of 0 hours (IQR 0.0, 1,2) in the standard care group and 3.3 hours (IQR 2.1, 4,7) in APP group.

**Table 1.** Baseline characteristics of patients.

	Standard Care		Prone Position	
	n	Summary statistic	n	Summary statistic
Age	47	66 (42, 76)	46	64 (44, 73)
Female Sex	47	17 (36%)	46	15 (33%)
BMI (kg/m2)	47	23 (20, 25)	46	24 (21, 26)
Hypertension	47	31 (66% )	46	26 (57%)
Diabetes	47	18 (38%)	46	19 (41%)
HIV/AIDS	47	14 (30%)	46	11 (24%)
Duration of symptoms on admission to hospital (days)	47	5 (2, 8)		4 (2, 7)
Previous vaccination	47	44 (94%)	46	41(89%)
Steroids on admission to hospital	47	42 (89%)	46	43 (93%)
Respiratory support at enrolment HFNC Nasal/Mask	47		46	
		19 (40%)		14 (30%)
		28 (60%)		32 (70%)
SOFA score	47	2 (1,3)	46	1 (1,3)
APACHE II score	47	12 (8,15)	46	10 (7,13)
Charleston Comorbidity Score	47	2 (1,6)	46	1 (0,6)

n = number of patients included in that summary statistic. - Values in the form of X (A, B) are medians followed by the 25th and 75th percentiles in parentheses.

**Figure 1.**
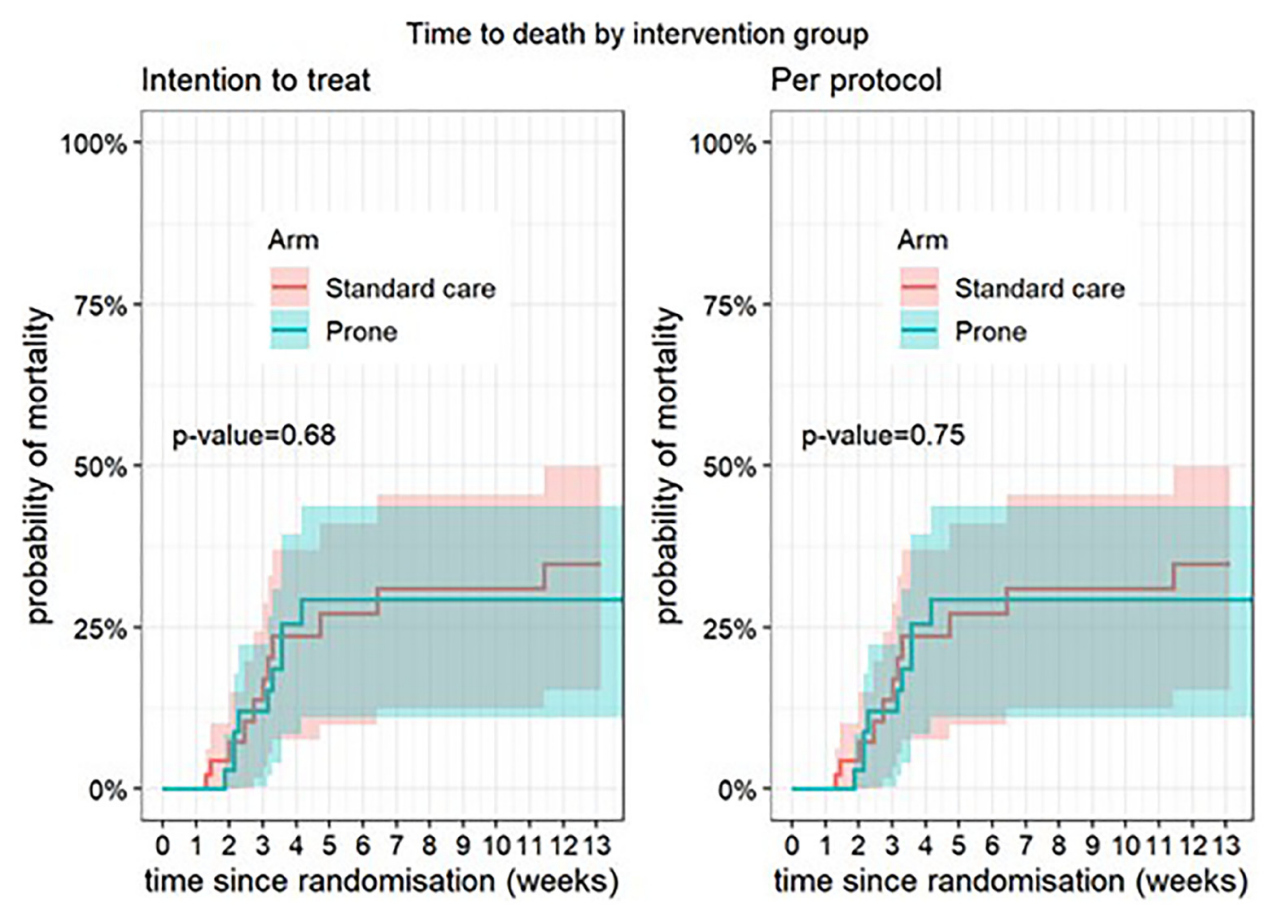
Time to death according to intervention group – intention to treat population.

There was no difference in the primary outcome of escalation of respiratory care within 28 days (RR 0.85, 95% CI 0.40, 1.78, p=0.67) ( Table 2). For the secondary outcomes of requirement for intubation and mechanical ventilation, 28-day mortality, in-hospital mortality, ventilator-free days, and duration of oxygen therapy, there was no difference between those allocated to APP or standard care ( Table 3). Similarly, we detected no changes in SpO _2_, respiratory rate, heart rate, or ROX index before and after prone sessions ( Table 3, Table 4 and Figure 1– Figure 8).

**Table 2.** Primary outcome (intention to treat and per protocol population).

	Sample			Regression		
	N	Non-escalation ^1^	Escalation ^1^	RR ^2^	95% CI ^2^	p-value
**Intention to treat**	93					
Standard care	47	35 (74%)	12 (26%)	—	—	—
Prone	46	36 (78%)	10 (22%)	0.85	0.40, 1.78	0.67
**Per protocol**	90					
Standard care	46	34 (74%)	12 (26%)	—	—	
Prone	44	34 (77%)	10 (23%)	0.87	0.41, 1.82	0.71

^1^n (%) ^2^RR = Relative Risk, CI = Confidence Interval

**Table 3.** Secondary outcomes, intention to treat population.

	Standard care		APP		Outcome measure		
	n	Summary statistic	n	Summary statistic		95% CI	P value
Intubation and MV within 28 days n (%)	47	8 (17%)	46	6 (13%)	RR 0.77	0.27, 2.04	0.59
28-day mortality n(%)	47	8 (17%)	46	8 (17%)	RR 1.02	0.41, 2.56	0.96
In-hospital mortality n(%)	47	11 (23%)	46	10 (22%)	RR 0.93	0.43, 2.00	0.85
Duration hospital stay (days) (median, IQR)	47	12 (10, 20)	46	11 (9, 16)	Beta -1.72	-8.15, 4.72	0.60
Duration of oxygen therapy ^1^ (days) median (IQR)	47	6 (3,11)	46	5 (3, 9)	Beta -1.32	-3.48, 0.85	0.23
Ventilation free days ^2^ (days) median (IQR)	47	11 (9,16)	46	10 (8, 15)	Beta -2.3	-4.8, 0.2	0.07

1. Supplemental oxygen via low flow nasal canulae/mask or HFNC 2. Ventilation free days until hospital discharge

**Table 4.** Secondary outcomes, per protocol population.

	Standard care		Prone		Outcome measure		P value
	n	Summary Statistic	n	Summary Statistic		95% CI	P value
Intubation and MV within 28 days n(%)	46	8 (17%)	44	6 (14%)	RR 0.78	0.28, 2.08	0.62
28-day mortality n(%)	46	8 (17%)	44	8 (18%)	RR 1.05	0.42, 2.61	0.92
In-hospital mortality n(%)	46	11 (24%)	44	10 (23%)	RR 0.95	0.44, 2.04	0.89
Duration hospital stay (days) (median, IQR)	46	13 (10, 21)	44	11 (9, 16)	Beta -1.73	-8.36, 4.91	0.61
Duration of oxygen therapy ^1^ (days) (median, IQR)	46	6 (3,7)	44	5 (3,6)	Beta -1.25	-3.48, 0.97	0.27
Ventilator free days ^2^ (median, IQR)	46	11 (9,17)	44	9 (8,15)	Beta -2.41	-5.0, 0.2	0.07

1. Supplemental oxygen via low flow nasal canulae/mask or HFNC 2. Ventilation free days until hospital discharge

**Figure 2.**
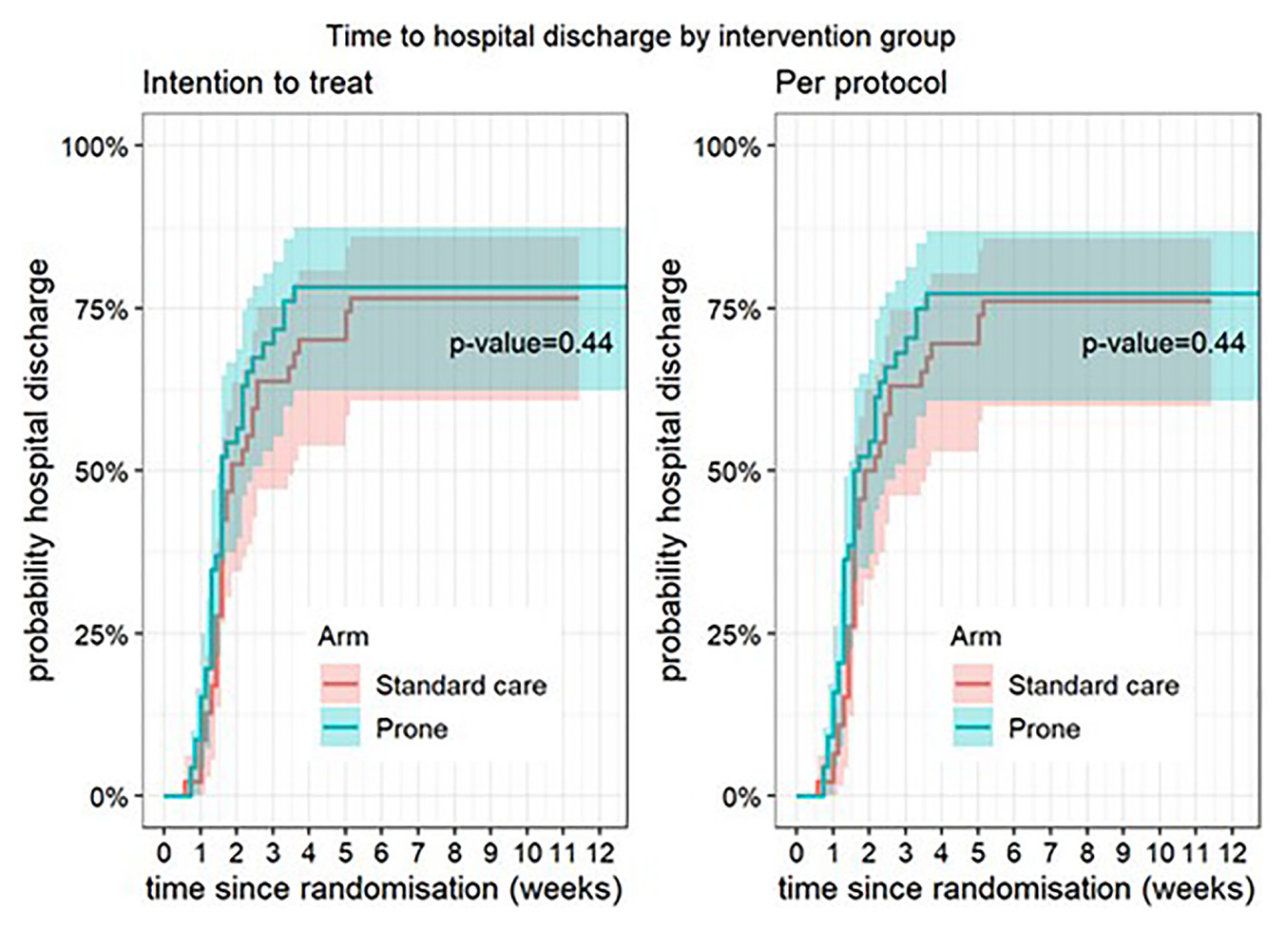
Time to hospital discharge (intention-to-treat and per-protocol populations). The P-value relates to cause-specific cumulative incidence tested using Gray’s log-rank test.

**Figure 3.**
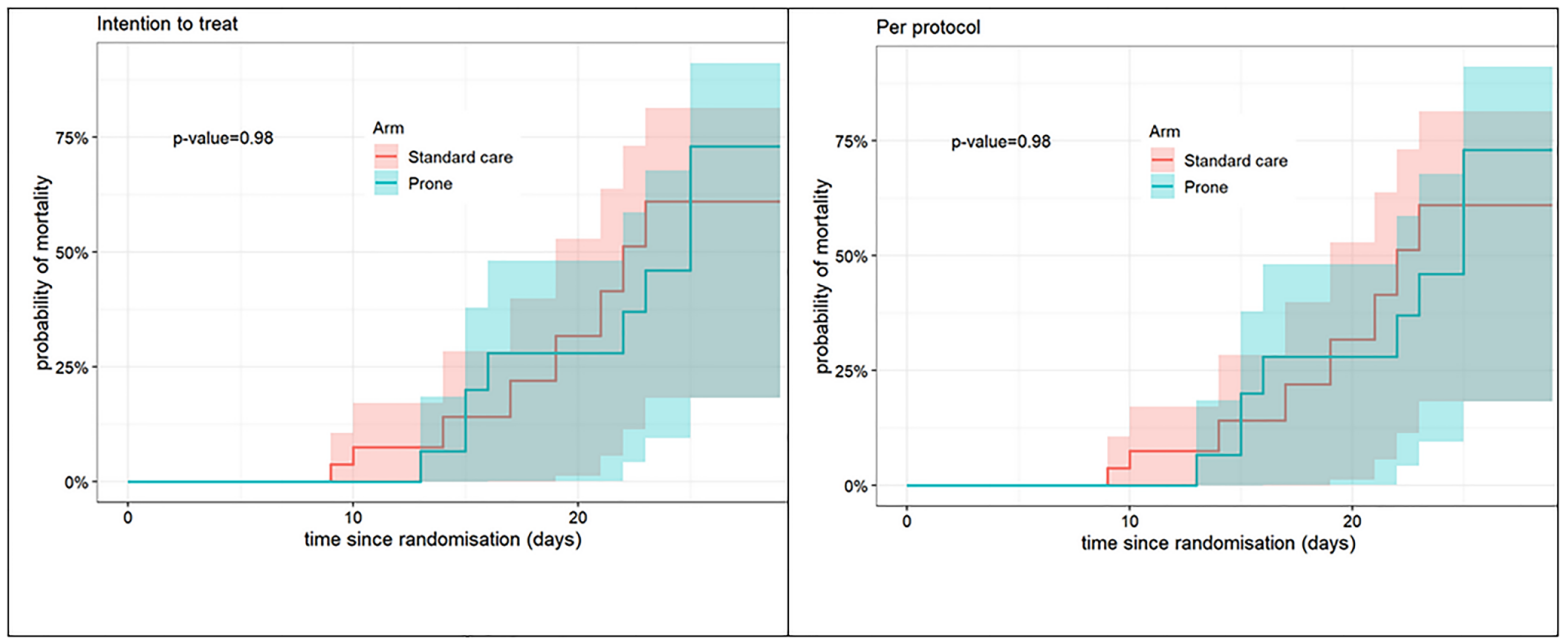
Kaplan Meier Curves for mortality 28-day all-cause mortality (intention-to-treat and per-protocol populations). The P-value relates to cause-specific cumulative incidence tested using Gray’s log-rank test.

**Figure 4.**
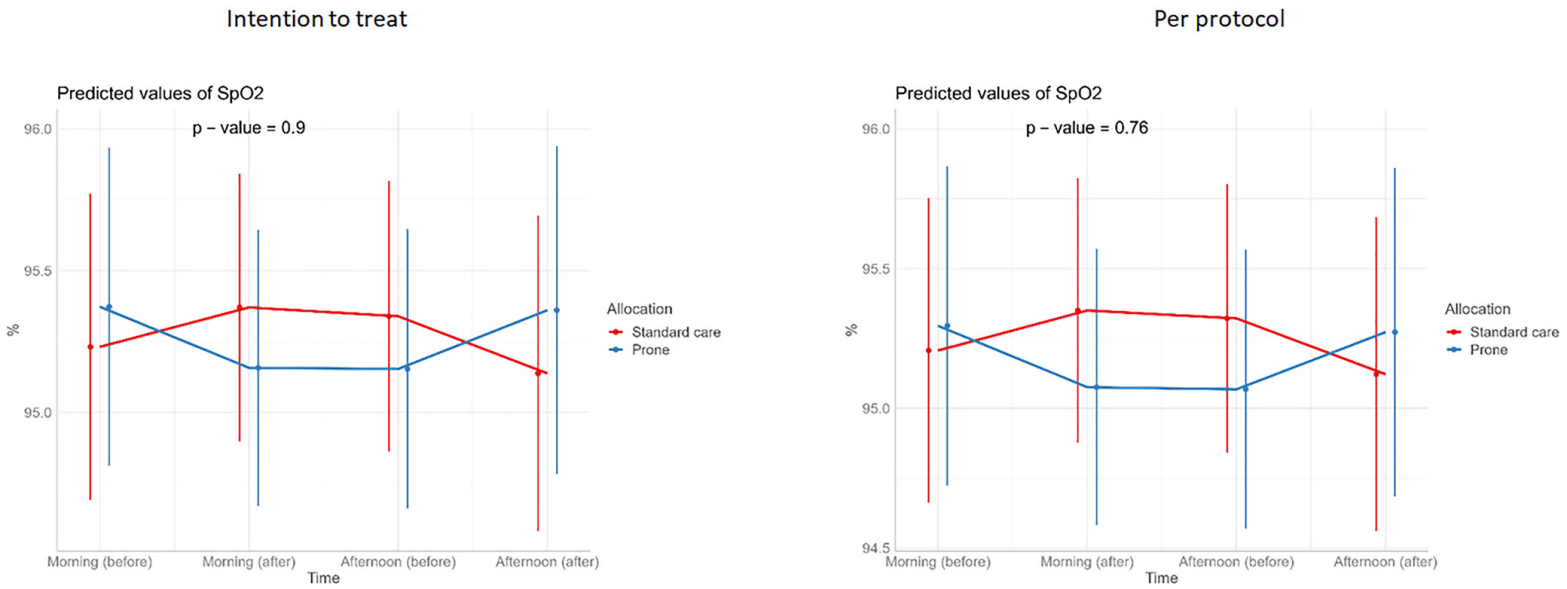
Longitudinal vital signs parameters: SpO _2_ (intention-to-treat and per-protocol populations). The p-values refer to the overall effect of intervention on the outcome.

**Figure 5.**
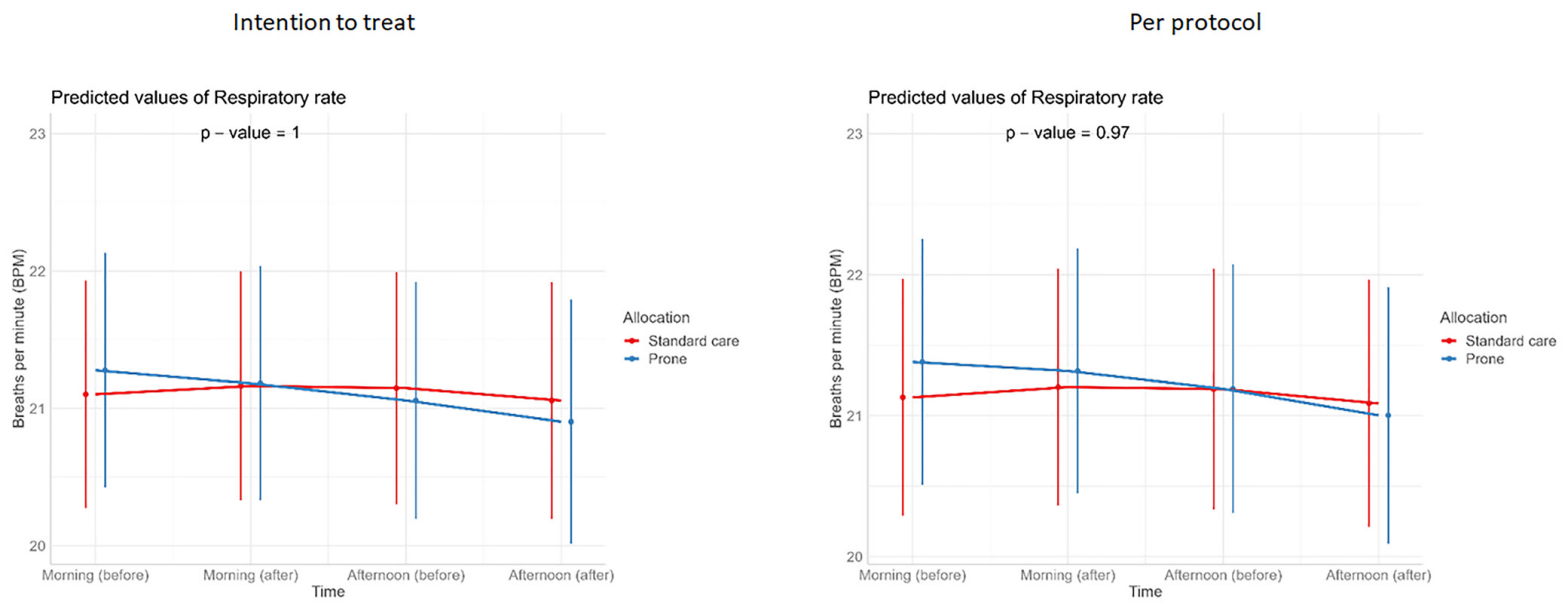
Longitudinal vital signs parameters: respiratory rate (intention-to-treat and per-protocol populations). The p-values refer to the overall effect of intervention on the outcome.

**Figure 6.**
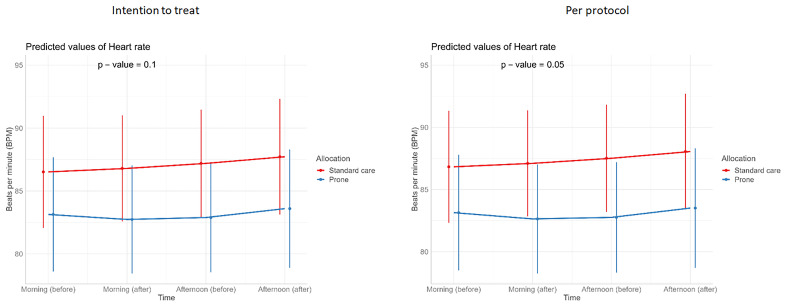
Longitudinal vital signs parameters: heart rate (intention-to-treat and per-protocol populations). The p-values refer to the overall effect of intervention on the outcome.

**Figure 7.**
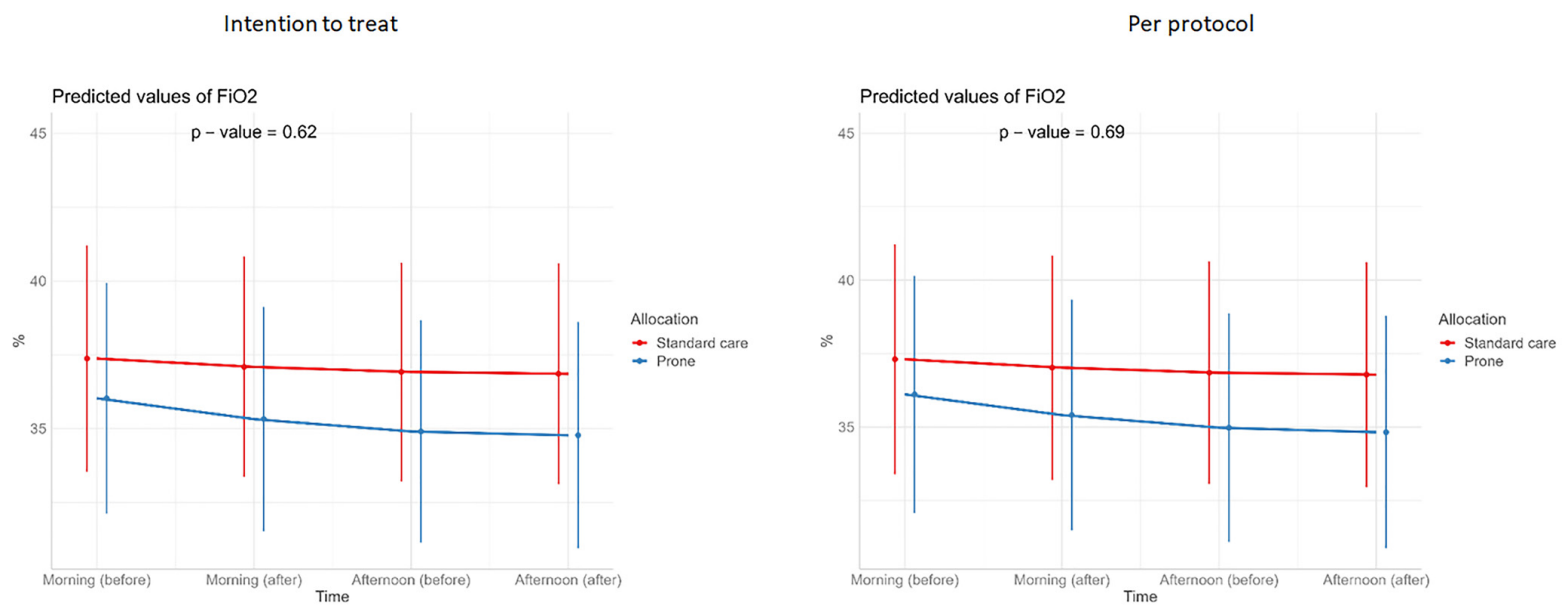
Longitudinal vital signs parameters: FiO _2_ (intention-to-treat and per-protocol populations). The p-values refer to the overall effect of intervention on the outcome.

**Figure 8.**
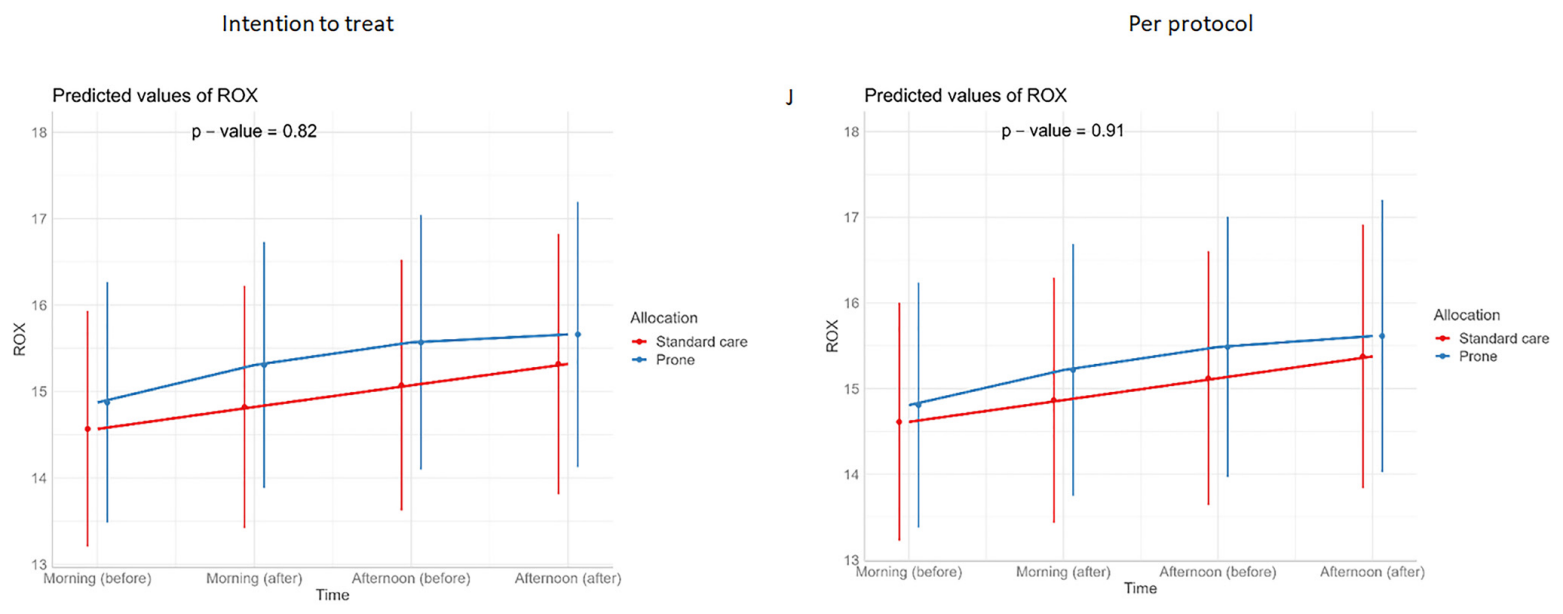
Longitudinal vital signs parameters: ROX (intention-to-treat and per-protocol populations). The p-values refer to the overall effect of intervention on the outcome.

None of the patients experienced adverse events attributed as definitely related to the intervention. There was no difference in adverse events judged as possibly related to the intervention groups (10/46 (24%) in the standard care group and 8/44 (18%) in the APP group). These events were pneumonia or exacerbation of chronic pulmonary disease ( Table 5). Severe adverse events occurred in 9/44 (20%) patients in the prone care group and 12/46 (26%) patients in the standard care group (p=0.56) ( Table 6). None of these serious adverse events was judged to be related to or possibly related to the intervention.

**Table 5.** Adverse events, per protocol population.

	Prone (N=44)		STDCARE (N=46)		
Characteristic	n	Summary statistic	n	Summary statistic	
Definitely Related AE	44	0/44 (0%)	46	0/46 (0%)	-
Possibly Related AE	44	8/44 (18%) *	46	10/46 (22%) **	0.673
Number of AEs per patient	44		46		0.624
- 0		7/44 (16%)		3/46 (7%)	
- 1		4/44 (9%)		5/46 (11%)	
- 2		3/44 (7%)		7/46 (15%)	
- 3		6/44 (14%)		6/46 (13%)	
- 4		6/44 (14%)		5/46 (11%)	
- 5		5/44 (11%)		3/46 (7%)	
- >5		13/44 (30%)		17/46 (37%)	

* 7 pneumonia, 1 blood stream infection/pneumonia. ** 10 pneumonia or exacerbation of chronic pulmonary disease.

**Table 6.** Severe adverse events, per protocol population.

	Prone (N=44)		Standard care (N=46)		
Characteristic	n	Summary statistic	n	Summary statistic	
Severe Adverse Event	9		12		0.56
- Cardiopulmonary Failure		0/9 (0%)		1/12 (8%)	
- Multi organ failure, shock		6/9 (67%)		6/12 (50%)	
- Multiorgan failure		2/9 (22%)		2/12 (17%)	
- Septic shock		1/9 (11%)		3/12 (25%)	

### Wearable device data

Data from wearable devices were available for all patients in whom devices had been used (45 in standard care and 44 in APP). Exploratory analyses showed increased monitoring data available from those in the standard care group linked to a longer duration of monitoring in those in the standard care group. Patients monitored in the standard care group had gyrometry recordings for a median of 118 hours (IQR 73, 187) compared to 93 hours (IQR 70,171) in the APP group. The mean daily prone hours recorded were 4.3 hours (IQR 1.9, 7.4) in the standard care group compared to 7.3 hours (IQR 4.3, 9.2) in the APP group (p=0.006).

### Patient perspectives

Patients’ perspectives on the APP revealed that most patients found APP comfortable. Similarly, they expressed general ease in entering and exiting the prone position ( Table 7). Nevertheless, 29/43 (70%) patients in the APP group preferred to be in a supine position during the day and 23/43 (55%) preferred to be supine at night, which was similar to the preferences reported by the standard care group. Patients reported that the wearable monitoring devices were very comfortable (median scores of 8 (7–9) and 9 (8–9) out of 10 in the standard care and prone groups, respectively) ( Table 7).

**Table 7.** Questionnaire Results.

	Standard Care (N=42)		Prone (N=43)	
Comfort of prone position (0-10 scale, with 0 extremely uncomfortable and 10 extremely comfortable), median (1 ^st^ and 3 ^rd^ quartile)	n=21	8 (6, 8)	n=42	7 (6, 8)
Ease of getting in and out of prone position (0-10 scale, with 0 very difficult and 10 very easy), median (1 ^st^ and 3 ^rd^ quartile)	n=21	8 (5.5, 9)	n=42	8 (6, 8)
Comfort of wearable devices ((0-10 scale, with 0 extremely uncomfortable and 10 extremely comfortable), median (1 ^st^ and 3 ^rd^ quartile)	n=43	8 (7, 9)	n= 42	9 (8, 9.25)
Preferred daytime position whilst receiving oxygen n (%) Supine Prone Side Sitting up No preference	n=43	30 (70) 3 (7) 6 (14) 4 (9) 0	n=42	29 (70) 3 (7) 8 (19) 2 (5) 0
Preferred nighttime position whilst receiving oxygen n( %) Supine Prone Side Sitting up No preference	n=43	24 (56) 1 (2) 18 (42) 0 (0) 0 (0)	n=42	23 (55) 2 (5) 16 (38) 0 (0) 1 (2)

## Discussion

Our study was terminated early due to a significant reduction in the number of patients hospitalized with COVID-19, reflecting the overall trend of the pandemic and the success of the vaccination program in Vietnam. Despite failing to reach the required sample size, there are several important findings and conclusions.

Our final sample size was underpowered to detect anticipated differences in outcomes, and our study outcome rate was lower than expected, perhaps due to the vaccination status of our population and routine use of steroid treatment. However, we did not detect significant differences in the primary or secondary outcome measures. Nevertheless, we did not observe any evidence of harm from the intervention. Importantly, in comparison to Qian *et al.*, ^4^ who reported high oxygen dependence at 5 days in patients treated with APP in a non-randomized clinical trial, we observed a trend towards shortening supplemental oxygen therapy (nasal, mask, or HFNC) in the APP group. This is further supported by the reduced monitoring hours in the APP group, as monitors were removed when the patients no longer required oxygen therapy.

The difference we observed in the primary outcome between groups is similar to that reported in the large meta-analysis ^3^, and data from our study will contribute to further analyses. Our study protocol and endpoints were designed to allow this. Of note, our study is the first in a vaccinated population, and the similarity in behavior of this population with previously studied unvaccinated patients underlines the validity of our findings.

Importantly, our study has demonstrated that achieving a significant duration of prone positioning in our population was extremely challenging, an important finding for policymakers in LMICs, such as Vietnam. Despite dedicated study staff available in the ward, our average prone duration was less than our target of 8 hours a day. The reasons for this included frequent interruptions to prone positioning due to routine ward care, mealtimes, and general frailty in our study population, who required significant help to turn prone. We demonstrated that an APP is not a resource-free intervention. Staff are needed to communicate with patients, assist them in achieving and maintaining the prone position, and monitor for potential adverse events. 

The use of wearable devices and dedicated study staff allowed us to accurately quantify the time patients spent in the prone position. We note that accelerometer data indicate that patients in both the standard and APP groups changed positions frequently. The discrepancy between the accelerometer-recorded prone position and that observed by our study staff is likely due to several reasons. First, accelerometer data include unobserved night-time movements of patients and second, our accelerometer data is the sum of 30 second intervals, thus it may include many short periods of prone not accounted for by ward staff. Whilst our choice of the infraclavicular fossa for sensor positioning aimed to reduce false indication of position changing and focused our analysis on the position of the thorax, accelerometer data used arbitrary cut-offs and therefore thoracic positioning, not seen as ‘prone’ by observers may be classified as prone, for example side-lying.

We have demonstrated that applying technologies such as wearable devices may allow detailed monitoring despite limited staff availability. The lack of routine electronic health record data and monitoring is a major impediment to performing high-quality clinical trials in LMICs, without significant investment in trial staff and infrastructure. The use of novel technologies can potentially eliminate such barriers to participation, redress inequity in research, and bias in data.

Our study improves the evidence base for APP as an intervention for COVID-19 patients. Data will be included in an ongoing meta-analysis, emphasizing the value of harmonized outcome data and the methodology used in this and other studies. It remains unclear whether these findings can be translated to the treatment of pneumonia due to other causes. Simple and effective means of improving the outcomes of patients with these infections could have important consequences for patients and resource utilization.

## Declarations

### Ethics and consent

The study protocol was approved by the Ethics Committee of the Hospital for Tropical Diseases (23/12/21) and Oxford Tropical Research Ethics Committee (Ref 39-21 (3/12/21). All participants or their representatives provided written informed consent before enrolment in the study.

## Data Availability

Data is available through a managed access policy at Oxford University Clinical Research Unit. Application to the data access committee is detailed on our website. Figshare: CONSORT checklist and flowchart for Awake prone positioning effectiveness in moderate to severe COVID-19 a randomized controlled trial. Combined consort checklist and flow diagram 06NV.pdf’. https://doi.org/10.6084/m9.figshare.26830342 ^22^ Data are available under the terms of the Creative Commons Zero "No rights reserved" data waiver (CC0 1.0 Public domain dedication).
