# Supplementary material for: Awake prone positioning effectiveness in moderate to severe COVID-19 a randomized controlled trial
Source: Wellcome Open Res. 2024 Dec 7;9:543. Originally published 2024 Sep 20. [Version 2] doi: 10.12688/wellcomeopenres.22792.2 (PMC11626102; doi:10.12688/wellcomeopenres.22792.2)
Supplement: Peer review history

# Reviewer report: Reviewer response for version 2

**DOI:** 10.21956/wellcomeopenres.25910.r116347

**Published:** 2025-01-17

**Authors:** Florence Daviet

**Affiliations:** 1 Médecine Intensive Réanimation, Marseille, France

This is a randomized controlled trial which aimed to evaluate the efficacy of APP in patients with moderate to severe COVID-19 in a tertiary level hospital in Vietnam. With the limit of not being able to reach the appropriate number of patients to include, the authors do not show a difference on the primary outcome (escalation of respiratory support within 28 days). This study highlights  also the difficulties to effectively implement APP, with majority of patient not reaching the target of 8h/day of APP despite a dedicated team to help them.

 The study is well conducted and the paper clearly written. I have no major concern.

**Some minor concerns are :**

 - discussion could detail more precisely the comparison with the results from previous meta analysis.

 -patients included are not very severe (FiO2 between 35 and 40% in figure 7), limiting the extrapolability of the results, as for the monocentric nature of the study.

Is the work clearly and accurately presented and does it cite the current literature?

Yes

If applicable, is the statistical analysis and its interpretation appropriate?

Yes

Are all the source data underlying the results available to ensure full reproducibility?

Yes

Is the study design appropriate and is the work technically sound?

Yes

Are the conclusions drawn adequately supported by the results?

Yes

Are sufficient details of methods and analysis provided to allow replication by others?

Yes

Reviewer Expertise:

My area of research is ICU, ARDS of any cause, including COVID 19 ARDS

I confirm that I have read this submission and believe that I have an appropriate level of expertise to confirm that it is of an acceptable scientific standard.

# Reviewer report: Reviewer response for version 2

**DOI:** 10.21956/wellcomeopenres.25910.r114921

**Published:** 2024-12-09

**Authors:** Haibo Qiu

**Affiliations:** 1 Southeast University, Nanjing, China

The authors have made a commendable revision to the manuscript. I have no additional comments.

Is the work clearly and accurately presented and does it cite the current literature?

Yes

If applicable, is the statistical analysis and its interpretation appropriate?

I cannot comment. A qualified statistician is required.

Are all the source data underlying the results available to ensure full reproducibility?

Yes

Is the study design appropriate and is the work technically sound?

Partly

Are the conclusions drawn adequately supported by the results?

Yes

Are sufficient details of methods and analysis provided to allow replication by others?

Partly

Reviewer Expertise:

critical care medicine, mechanical ventilation, ARF

I confirm that I have read this submission and believe that I have an appropriate level of expertise to confirm that it is of an acceptable scientific standard.

# Reviewer report: Reviewer response for version 1

**DOI:** 10.21956/wellcomeopenres.25097.r111143

**Published:** 2024-12-08

**Authors:** Luke A McDonald

**Affiliations:** 1 Austin Health, Melbourne, Australia

This is a well designed study that aims to assess the efficacy and acceptability of awake prone positioning in patients with COVID-19 requiring supplemental oxygen. 

 The trials primary outcome - escalation of respiratory therapy within 28 days of randomisation - is of clinical relevance in this population and setting. 

 Due to widespread vaccination uptake in Vietnam, there was a substantial reduction in eligible patients and accordingly, the study was ceased early without reaching the calculated sample size. It is important to note that ceasing this trial early does not reflect poor trial design. 

 The authors have appropriately interpreted the results from the reduced sample, without overstating any findings. 

 Whilst a small study, as discussed by the authors, the results may be included in a meta-analysis of awake prone positioning to further strengthen the evidence base.

Is the work clearly and accurately presented and does it cite the current literature?

Yes

If applicable, is the statistical analysis and its interpretation appropriate?

Yes

Are all the source data underlying the results available to ensure full reproducibility?

Yes

Is the study design appropriate and is the work technically sound?

Yes

Are the conclusions drawn adequately supported by the results?

Yes

Are sufficient details of methods and analysis provided to allow replication by others?

Yes

Reviewer Expertise:

Critical Care, Respiratory Medicine, Prone Positioning, Physiotherapy

I confirm that I have read this submission and believe that I have an appropriate level of expertise to confirm that it is of an acceptable scientific standard.

# Reviewer report: Reviewer response for version 1

**DOI:** 10.21956/wellcomeopenres.25097.r111149

**Published:** 2024-11-26

**Authors:** Haibo Qiu

**Affiliations:** 1 Southeast University, Nanjing, China

Thanks for giving me this chance to review this trial. Since COVID-19 pandemic, APP has been widely used in the clinical practice. Despite several trial has been conducted to explore the efficacy of APP, it is not completely clear. The authors performed a single, randomized trial to evaluate the efficacy and acceptability of APP in a lower-middle income country. They found that APP did not reduce the escalation of respiratory support within 28 days. Please see my major and minor comments below and I hope these can be used to help to strengthen the manuscript.

**Major:**

1. The duration of APP may affect its efficacy. However, the target duration of APP in the APP group was lack. Can you please explain the reason?
2. The intervention in the APP group is not very clear. Could you please add the detailed intervention method?
3. The sample size calculation may not be suitable. The primary outcome is escalation of respiratory therapy within 28 days of randomization, while the sample size was calculated based on a treatment failure rate; there was lack of absolute change in the APP group; why did you not consider the dropout rate?

**Minor:**

1. Introduction, the background and the aim of this study are not closely related. Please focus on the aim of the study.
2. Can you add the flowchart of study participants in the manuscript?
3. Baseline SpO _2_/FiO _2_ is lack. Please add this information in the Table 1.
4. Please present the duration of APP in both groups.
5. The secondary outcomes did not require analysis in the per-protocol population.

Is the work clearly and accurately presented and does it cite the current literature?

Yes

If applicable, is the statistical analysis and its interpretation appropriate?

I cannot comment. A qualified statistician is required.

Are all the source data underlying the results available to ensure full reproducibility?

Yes

Is the study design appropriate and is the work technically sound?

Partly

Are the conclusions drawn adequately supported by the results?

Yes

Are sufficient details of methods and analysis provided to allow replication by others?

Partly

Reviewer Expertise:

critical care medicine, mechanical ventilation, ARF

I confirm that I have read this submission and believe that I have an appropriate level of expertise to confirm that it is of an acceptable scientific standard, however I have significant reservations, as outlined above.

# Response

**Published:** 2024-12-01

**Authors:** C Louise Thwaites

**Affiliations:** Oxford University Clinical Research Unit, Vietnam

- Thank you for reviewing our article and your questions are very much ones we have considered already in carrying out the study and preparing the manuscript.
- The duration of APP is indeed important and was the rationale for us supervising the APP periods to try and enable this to be as long as possible. The reasons for lack of APP reaching the appropriate timing varied but were generally reported as patients being otherwise engaged (eating, washing), discomfort, inability to turn themselves. These are included in the discussion section as they were not formally collected in our results or analysis plan: “ *The reasons for this included frequent interruptions to prone positioning due to routine ward care, mealtimes, and general frailty in our study population, who required significant help to turn prone.”*
- The description of APP in detail is given in the supplementary materials as the format of this publication and the editorial committee did not allow Appendices. We have supplemented the reference to these supplementary materials with the DOI index where these can be found. In addition the methods section contains the following information:
-  “Patients in the APP group were visited by the study team and provided written and verbal advice about lying in the prone position, as well as assistance in achieving and maintaining a fully prone position for as long as possible. All study procedures were carried out by a specific study team who were present in the ward at 8am-5pm daily and dedicated ward nurses who supervised the evening APP session (6–8pm). APP was initiated as soon as possible after randomization and continued until either escalation or cessation of oxygen therapy. Patients in the APP group were supported to be in the prone position for as long as possible while the study staff were attending the ward, except for mealtimes or other nursing procedures. Support included both physical assistance and physical aid such as pillows. No support was available to assist patients in turning prone at night time after 8pm.”
- A dropout rate was included in the sample size estimation (performed by a biomedical statistician). However as the sample size was not reached due to the decision to halt the trial early. In reality this rate was very low. We have modified the text to show that drop-out and protocol violations were indeed included in this sample size calculation.
- We have made our aim more clear – in that we aimed to evaluate APP of longer duration (8 hrs a day), using a dedicated team to achieve this. We feel that this now is appropriate for our introduction. This includes what is known about prone position (largely data in MV patients), what is already known about APP in COVID-19 and the limitations of these studies, particularly with relevance to a LMIC setting and where our study aims to fit in terms of knowledge gap.
- To address your point we have emphasized the points around the aim of the study further in the introduction section.
- The editorial guidelines have not permitted us to include a Flowchart in this publication. We have therefore included this in the Additional materials. Thank you for drawing attention to this point as we had asked to be able to include this. This is available at the following point, as detailed in the manuscript.
- Our analysis and results presentation have followed our a-priori statistical analysis plan. As the use of nasal canulae, low-flow and high-flow systems means that an accurate SpO2/FiO2 could be highly misleading. For that reason we elected at this point to include only the level of respiratory support as a guide to the severity of patients. This is consistent with other published studies of awake prone positioning in this population and makes this more comparable. For our results, we have analysed the change in ROX index, but these considerations still apply to this population.
- Duration of APP is presented already using both methods in the results section and included in the discussion section. Mean of daily APP duration per individual observed by study staff was a median of 0 hours (IQR 0.0, 1,2) in the standard care group and 3.3 hours (IQR 2.1, 4,7) in APP group. The mean daily prone hours recorded were 4.3 hours (IQR 1.9, 7.4) in the standard care group compared to 7.3 hours (IQR 4.3, 9.2) in the APP group (p=0.006).
- We have included secondary outcomes per-protocol analyses for completeness and for transparency. We maintain that intention to treat analysis is primarily presented. Again we are restricted by the journal’s policy on publishing these in the main manuscript as opposed to a supplement.

# Reviewer report: Reviewer response for version 1

**DOI:** 10.21956/wellcomeopenres.25097.r103719

**Published:** 2024-10-18

**Authors:** Giorgio Maraziti

**Affiliations:** 1 Internal Vascular and Emergency medicine - Stroke Unit, Azienda Ospedaliera di Perugia (Ringgold ID: 18633), Perugia, Umbria, Italy

This randomized controlled trial evaluated the efficacy and acceptability of awake-prone positioning (APP) in patients with moderate to severe COVID-19 in a lower-middle-income setting. The trial was terminated early due to reduced COVID-19 admissions; as a result, it did not reach its planned sample size of 300 patients per arm. It showed no significant differences between the APP group and standard care regarding respiratory support escalation, intubation rates, or mortality. Additionally, APP was found to be safe, but achieving the targeted prone time of 8 hours was challenging, and most patients preferred supine positioning. The use of wearable monitoring devices to control APP was feasible and provided valuable data.

 The work is clearly and accurately presented and cites the current literature. The randomization process and statistical analysis are adequately described. The results are consistent with the data, and the conclusions have been drawn adequately. I have no significant concerns about this study.

**I have some minor comments:**

**Background section:**

- It may be better to cite more references related to the following sentence: "The COVID-19 pandemic stimulated multiple randomized controlled trials of prone positioning in non-mechanically ventilated patients."
- The acronym LMIC was first introduced and needs to be explained.

**Methods:**

- Body mass index > 35 needs a unit (kg/m²).
- Were sedative drugs used to facilitate the APP? Given APP’s very short mean time of 3.3 hours versus the target of 8 hours in the APP group, I suppose not; however, I think this doubt should be addressed. Moreover, the use of sedative drugs or other procedures to facilitate the maintenance of APP may be discussed in the Discussion section.

Is the work clearly and accurately presented and does it cite the current literature?

Yes

If applicable, is the statistical analysis and its interpretation appropriate?

Yes

Are all the source data underlying the results available to ensure full reproducibility?

Yes

Is the study design appropriate and is the work technically sound?

Yes

Are the conclusions drawn adequately supported by the results?

Yes

Are sufficient details of methods and analysis provided to allow replication by others?

Yes

Reviewer Expertise:

emergency medicine, respiratory failure, pulmonary embolism, stroke

I confirm that I have read this submission and believe that I have an appropriate level of expertise to confirm that it is of an acceptable scientific standard.
